# Procalcitonin-guided antibiotic use versus a standard approach for acute respiratory tract infections in primary care: study protocol for a randomised controlled trial and baseline characteristics of participating general practitioners [ISRCTN73182671]

**DOI:** 10.1186/1471-2296-6-34

**Published:** 2005-08-18

**Authors:** Matthias Briel, Mirjam Christ-Crain, Jim Young, Philipp Schuetz, Peter Huber, Pierre Périat, Heiner C Bucher, Beat Müller

**Affiliations:** 1 Basel Institute for Clinical Epidemiology, University Hospital Basel, CH-4031 Basel, Switzerland; 2 Clinic of Endocrinology, Diabetes & Clinical Nutrition, Department of Internal Medicine, University Hospital Basel, CH-4031 Basel, Switzerland; 3 Department of Chemical Pathology, University Hospital Basel, CH-4031 Basel, Switzerland; 4 General practice, In den Neumatten 63, CH-4125 Riehen, Switzerland

## Abstract

**Background:**

Acute respiratory tract infections (ARTI) are among the most frequent reasons for consultations in primary care. Although predominantly viral in origin, ARTI often lead to the prescription of antibiotics for ambulatory patients, mainly because it is difficult to distinguish between viral and bacterial infections. Unnecessary antibiotic use, however, is associated with increased drug expenditure, side effects and antibiotic resistance. A novel approach is to guide antibiotic therapy by procalcitonin (ProCT), since serum levels of ProCT are elevated in bacterial infections but remain lower in viral infections and inflammatory diseases.

The aim of this trial is to compare a ProCT-guided antibiotic therapy with a standard approach based on evidence-based guidelines for patients with ARTI in primary care.

**Methods/Design:**

This is a randomised controlled trial in primary care with an open intervention. Adult patients judged by their general practitioner (GP) to need antibiotics for ARTI are randomised in equal numbers either to standard antibiotic therapy or to ProCT-guided antibiotic therapy. Patients are followed-up after 1 week by their GP and after 2 and 4 weeks by phone interviews carried out by medical students blinded to the goal of the trial.

Exclusion criteria for patients are antibiotic use in the previous 28 days, psychiatric disorders or inability to give written informed consent, not being fluent in German, severe immunosuppression, intravenous drug use, cystic fibrosis, active tuberculosis, or need for immediate hospitalisation.

The primary endpoint is days with restrictions from ARTI within 14 days after randomisation. Secondary outcomes are antibiotic use in terms of antibiotic prescription rate and duration of antibiotic treatment in days, days off work and days with side-effects from medication within 14 days, and relapse rate from the infection within 28 days after randomisation.

**Discussion:**

We aim to include 600 patients from 50 general practices in the Northwest of Switzerland. Data from the registry of the Swiss Medical Association suggests that our recruited GPs are representative of all eligible GPs with respect to age, proportion of female physicians, specialisation, years of postgraduate training and years in private practice.

## Background

Acute respiratory tract infections (ARTI) are among the most frequent reasons for seeking ambulatory care [1]. ARTI in the context of this study include common cold, pharyngitis, tonsillitis, rhinosinusitis, tracheo-bronchitis, otitis media, acute exacerbations of asthma and of chronic obstructive pulmonary disease (COPD), and community acquired pneumonia. As much as 75% of antibiotics are prescribed for ARTI, despite the mainly viral origin [2-8].

Criteria often used in clinical practice to distinguish bacterial from viral infections of the respiratory tract include fever, dyspnea, purulent sputum, chest X-ray infiltrates, C-reactive protein, leucocyte count, and recovery of a pathogen from the respiratory tract or from blood cultures [9]. However, these are all non-specific symptoms and hence differentiation between viral and bacterial ARTI remains a diagnostic challenge [10]. Moreover, when antibiotic treatment is initiated, the optimal duration of antibiotic treatment for ARTI has not been determined [11,12]. In community acquired pneumonia an antibiotic treatment duration of 10 to 14 days is generally recommended, although data from intervention trials are lacking [13].

Unnecessary antibiotic use (i.e. number of prescriptions and duration of treatment) for ARTI not only increases drug expenditure [14] and the risk of adverse events [15], but also results in selection of resistant microorganisms [16]. Thereby, it constitutes an important public health problem [17]. For combating the increase in resistant infections a decrease of the excess antibiotic use is paramount [18]. There are only few intervention studies that have reported a successful reduction of antibiotic use in ambulatory care [19-25]. Most of these studies were not conducted in ARTI or have methodological limitations.

A novel approach to guide antimicrobial therapy is to prescribe antibiotics based on the level of biomarkers, specifically, calcitonin precursors, including procalcitonin (ProCT). Circulating levels of ProCT are elevated in systemic bacterial infections but remain relatively low in viral infections and inflammatory diseases [26,27]. In severe bacterial infections the use of ProCT significantly improves the sensitivity and specificity of the clinical diagnosis of infection [28]. A recent systematic review and meta-analysis found that ProCT is superior compared to C-reactive protein for the diagnosis of bacterial infections [29]. Most recently, we gathered evidence that both antibiotic prescription and treatment duration could be safely and markedly reduced in hospitalised patients with lower respiratory tract infections using ProCT-stewardship [30,31]. In successfully treated infections, circulating ProCT levels decrease in a log-linear pattern and have a plasma half life of 24 hours. In contrast, prolonged elevated plasma ProCT levels indicate adverse outcome [26,27].

Several studies indicate that the main reasons for antibiotic prescription in ambulatory patients with ARTI are non-medical and related to the physician-patient relationship, patients' expectations and beliefs about the benefit of antibiotics [32,33]. Thus, in theory a reduction of antibiotic prescriptions and duration can also be achieved by the implementation of guidelines [34]. However, in practice physician education and guidelines dissemination for ARTI management usually show no clinically relevant effect [25,35,36].

## Methods/Design

### Study aims

The objective of this trial is to evaluate, if a ProCT-guided diagnostic and therapeutic strategy leads to a similar outcome and reduced total antibiotic use for patients with ARTI in primary care compared to a standard approach recommended by current guidelines.

### Study design and setting

**Figure 1:**
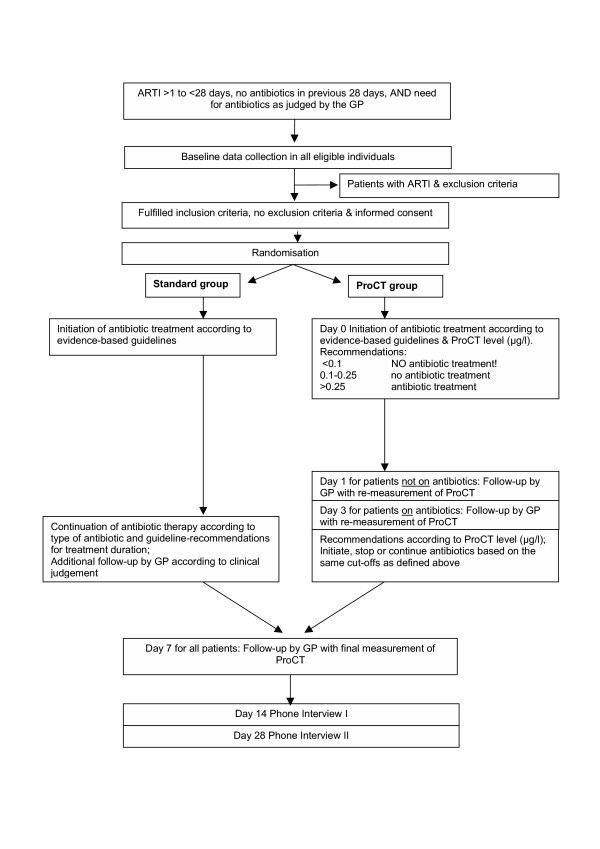
Summary of the trial design.

This is a prospective, randomised, controlled, open intervention trial in primary care with appropriate power calculation. Adult patients suffering from ARTI, for whom the treating general practitioner (GP) decides to give antibiotic treatment on the basis of evidence-based guidelines, are randomised to routine antibiotic therapy or ProCT-guided antibiotic treatment. The pathway by which patients are recruited and followed-up is given in Figure 1.

#### Ethical considerations

The Ethics Committee of the University Hospital Basel, Switzerland, approved the study protocol which is in compliance with the Helsinki Declaration. Written informed consent was obtained from all participating GPs. All recruited patients have to give written informed consent.

The trial is supervised by an independent monitoring board that is not involved in the design and conduct of the trial, or in the recruitment of patients. The board consists of a general internist in primary care, an infectious disease specialist and a pneumologist.

### Participants

**Figure 2:**
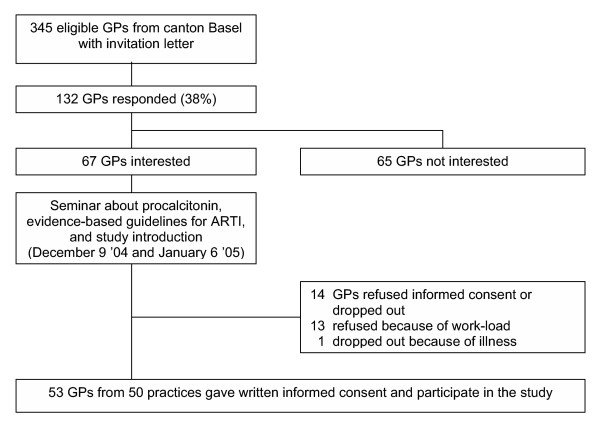
Flow diagram of recruited general practitioners.

We invited all GPs of two cantons (Basel-Stadt and Basel-Land) in the Northwest of Switzerland to participate in the trial. Of 345 GPs contacted, 53 working at 50 practices gave written informed consent and were included (Figure 2).

From December 2004 GPs included in this study consecutively screen all eligible adults (aged 18 years or older) with symptoms (first experienced within the previous 28 days) of acute infection of the respiratory system. Inclusion criteria for patients are a consultation for common cold, pharyngitis, tonsillitis, rhinosinusitis, tracheo-bronchitis, otitis media, influenza, acute exacerbations of asthma or COPD, or community acquired pneumonia, the GPs intention to prescribe antibiotics on the basis of evidence-based guidelines, and written informed consent. Exclusion criteria for patients are antibiotic use in the previous 28 days, intravenous drug use, psychiatric disorders or inability to give written informed consent, not being fluent in German, severe immunosuppression (e.g. in HIV-infection, after solid organ transplantation or under chemotherapy), cystic fibrosis, active tuberculosis, and need for immediate hospitalisation. Study practices complete the trial after including 20 patients or at the anticipated end of the trial in December 2005.

### Randomisation

Allocation of patients to either treatment group is concealed by using a centralised randomisation procedure with a computer generated list produced by an independent statistician otherwise not involved in the trial. Randomisation is stratified by GP practice.

### Interventions

All participating GPs received instructions about the protocol and the details of the clinical trial in a 1-hour seminar. They were asked to consecutively enrol all patients with ARTI that they judge to be in need of antibiotic treatment according to guidelines. They were told how to call the central randomisation unit, and how to fill in the necessary study forms.

#### Evidence-based guidelines

HCB and MB developed guidelines for the management of ARTI based on evidence-based US-position papers which were endorsed by the Centers for Disease Control and Prevention, the American Academy of Family Medicine, the American College of Physicians, and the Infectious Disease Society of America [37-42]. We systematically searched MEDLINE and the Cochrane Library to update this evidence with recent controlled clinical trials. A panel of local primary care providers, infectious disease experts, and clinical epidemiologists reviewed the guidelines and made suggestions for adaptation to local conditions. We distributed the guidelines as a booklet (see ) and presented them in a 2-hour seminar to all participating GPs.

#### Procalcitonin test

We measure ProCT by using a newly developed time-resolved amplified cryptate emission (TRACE) technology assay (Kryptor PCT, Brahms, Henningsdorf, Germany). This assay is based on a sheep polyclonal antibody against calcitonin and a monoclonal antibody against katacalcin, which bind to the calcitonin and katacalcin sequence of calcitonin precursor molecules. The assay has an improved functional assay sensitivity of 0.06 μg/l – i.e., three to ten fold above normal mean values. Assay time is 19 min with 20–50 μl of plasma or serum. The test is performed at the central laboratory of the University Hospital Basel, and results can be communicated to participating GPs within 2–4 h depending on the location of the practice.

### Data collection and management

We obtained baseline data on all eligible GPs from the registry of the Swiss Medical Association. These data suggested that included GPs are representative of all eligible GPs with respect to age, years in private practice, years of postgraduate training, years since diploma, specialisation, and percentage of female physicians (Table 1).

**Table 1:** Baseline characteristics of general practitioners

	**Study GPs**	All eligible GPs
	n = 53	n = 345

**Age **– median [IQR]	51 [42 – 55]	53 [47 – 58]
**Female physicians **– n (%)	9 (17)	64 (19)
**Specialisation**		
General medicine – n (%)	25 (47)	188 (54)
Internal medicine – n (%)	26 (49)	148 (43)
Other – n (%)	2 (3.8)	9 (2.6)
**Years in private practice **– median [IQR]	15 [6.2 – 20]	16 [9.0 – 23]
**Years of postgraduate training **– median [IQR]	8.8 [7.7 – 9.7]	8.9 [7.6 – 11]
**Years since diploma **– median [IQR]	25 [15 – 29]	26 [14 – 31]

When a participating GP intends to give antibiotic treatment to an eligible patient based on clinical criteria and the patient gives written informed consent, the GP calls the study centre and the patient is randomly allocated to one treatment group or the other. The GP then takes a blood sample from the patient and sends it by courier service to the laboratory of clinical chemistry at the University Hospital Basel. This laboratory measures ProCT in all patients. Additionally, the GP documents patient baseline data on signs and symptoms, diagnostic procedures, diagnosis, co-morbidity and prescribed medication.

Where patients are randomised to the ProCT-arm, GPs will be informed about ProCT results and given recommendations about appropriate antibiotic therapy within 2–4 h after the blood is taken depending on the location of the practice. A cut-off ProCT level of 0.1 μg/l is used to rule out a bacterial respiratory tract infection. This value is identical to the cut-off used for the evaluation of patients in the emergency department of the University Hospital Basel [30]. In patients with a ProCT level below 0.1 μg/l, the diagnosis of a bacterial respiratory tract infection is considered highly unlikely, and the GP is encouraged to look for viral or alternative causes. Accordingly, the use of antibiotics is discouraged. In patients with a ProCT level above 0.25 μg/l, a bacterial respiratory tract infection is considered the most likely diagnosis and the use of antibiotics is recommended. For ProCT levels from 0.1 to 0.25 μg/l, a bacterial infection is unlikely and antibiotic treatment is not advocated.

The GP then informs the patient about antibiotic treatment by phone. Patients in whom antibiotics are given will be asked to use a delayed prescription or to come back to the practice to pick up the antibiotic there. For patients in whom antibiotics are withheld based on ProCT levels of 0.25 μg/l or below, a follow-up measurement of ProCT within 24 hours is mandatory. If the ProCT level on this initial follow-up is >0.25 mg/l or if it has increased by more than 50% from its initial value without clinical improvement of the patient, the use of antibiotics is recommended.

In the ProCT group, all patients treated with antibiotics will be reassessed at Day 3. Discontinuation of antibiotic treatment is recommended if the ProCT level has decreased at least to 0.25 μg/l or below.

GPs draw blood samples and document therapy at each follow-up visit. They also collect information on days with restrictions and days off work at 1 week (6–8 days) after randomisation for all patients. Medical students, blinded to treatment allocation of patients and to the goal of the trial, will conduct standardised follow-up interviews at 14 and 28 days by phone. The patient flow will be monitored according to current guidelines and in agreement with the CONSORT statement [43]. We will use Teleform^® ^(Cardwell, Cardiff, GB) for data entry.

### Adverse events

Any serious adverse event is reported by fax to the principle investigator within 24 hours. We define a severe event independent of group allocation as hospitalisation for any reason, any complication related to infection such as sepsis, abscess etc., or allergic reaction due to the received therapy, or death that occurs within 28 days following the inclusion of the patient into the trial.

### Outcomes and hypotheses

The primary outcome is days with restrictions from ARTI within 14 days after randomisation. Secondary outcomes are antibiotic use in terms of antibiotic prescription rate and duration of antibiotic treatment in days, days off work and days with side-effects from medication within 14 days, and relapse rate from ARTI within 28 days after randomisation.

Our hypothesis is that the clinical outcome for patients with ARTI will be no worse under ProCT-guided treatment, but patients with ProCT-guided treatment will have lower total antibiotic use; specifically a 20% lower antibiotic prescription rate and a 20% shorter antibiotic duration compared to patients treated under the standard approach.

### Sample size considerations

This is a non-inferiority trial. We aim to show that on average ProCT-guided antibiotic management leads to at most one day more with restrictions than a standard approach. We consider a type I error rate of 5% and a type II error rate of 10% (i.e. 90% power) appropriate in this situation. In a previous trial in patients with acute respiratory tract infections (ISRCTN57824788), the standard deviation in the number of days with restrictions from ARTI was 4 days for those patients prescribed antibiotics. Given this previous estimate of the variability in the primary outcome, 275 patients are needed per treatment group [44]. Allowing for a loss to follow-up of 10% gives 306 patients per treatment group, or a total of 612 patients. This sample size will allow us to estimate the reduction in antibiotic use between the two arms to within ± 6%.

With a maximum of 20 patients recruited per practice we will probably need at least 35 participating general practices. To assess between-GP-variability in a sensitivity analysis, we will need a minimum of 10 patients (preferably 15) recruited per practice.

### Statistical analysis

With a non-inferiority trial, an intent-to-treat analysis is not necessarily conservative. For the primary outcome, we will need to provide several intent-to-treat analyses under different assumptions about patients who do not complete the trial, and a per-protocol analysis. Per-protocol analyses are planned for all secondary outcomes [44].

All outcomes will be analysed by a generalised linear model, assuming an appropriate distribution for each outcome and using the same set of covariates. These covariates will be: age, sex, education and a baseline score of the degree of restricted activity reported by the patient. 95% confidence intervals will be reported for the difference between treatment groups.

For the primary outcome, the assumed distribution will be normal; that is, analysis will be by multivariate linear regression. As a sensitivity analysis, the GP will be added to the model as a random effect and the model re-fit to the data from all GPs with only one GP per practice and with at least 10 patients per GP. Our experience from a previous trial (ISRCTN57824788) is that the difference between GPs has little influence on patient reported outcomes.

For secondary outcomes, assumed distributions will be binomial (i.e. for prescription of antibiotics) or normal (i.e. duration of antibiotic treatment). Duration of antibiotic treatment will be analysed only for those patients who receive antibiotics. We will also report a confidence interval for the difference in the antibiotic prescription rate between treatment groups. As a sensitivity analysis, we will repeat this calculation using a method appropriate for a cluster sample, where each GP forms a cluster and using only data from GPs with one GP per practice and with at least 10 patients per GP.

### Time plan for the study

Patient recruitment began in December 2004 and is planned to continue until December 2005. By April 2005, 213 patients (35% of target) have been recruited into the trial.

## Discussion

The present trial is the first randomised controlled trial to evaluate whether ProCT testing in a primary care setting reduces antibiotic use for ARTI without compromising patient relevant outcomes. In case of success, implementing this new approach into daily practice could largely improve the management of ARTI in primary care by avoiding unnecessary antibiotic use and preventing antibiotic resistance.

Our trial may have some limitations. First, this is an open intervention trial, and GPs may learn from their experience with ProCT testing and improve their clinical judgement. We cannot control for this bias, but at least this bias will be conservative for outcomes such as the antibiotic prescription rate and the duration of antibiotic therapy. Second, we expect to have recruited highly motivated primary care physicians, interested in the research question and able to provide high quality data. Motivated, interested GPs might be more reluctant to prescribe antibiotics for ARTI; thus they might consider patients for antibiotic treatment which are on average sicker than patients considered by disinterested GPs. However, we believe that ProCT-guided ARTI management will lead to a reduced antibiotic use even in such a setting of motivated GPs and the potential bias will be conservative. Third, while measurement of ProCT at a central laboratory is not ideal for routine primary care, there are still a considerable number of general practices that send blood samples daily to a laboratory for analysis of C-reactive protein or leucocytes. Therefore this should also be feasible for ProCT until a near-patient test, which is currently being developed, becomes widely available.

## Abbreviations

ARTI, acute respiratory tract infections

GP, general practitioner

ProCT, procalcitonin

## Competing interests

BM has served as consultant and received payments from BRAHMS (the manufacturer of procalcitonin assays) for speaking engagements, travel expenses, or research. The other authors declare that they have no competing interests.

## Authors' contributions

BM and HCB conceived of the study. All authors participated in the development of research protocols and in the design of the study. JY resolved statistical issues. MB drafted the manuscript. All authors read and corrected draft versions of the manuscript and approved the final manuscript.

## Pre-publication history

The pre-publication history for this paper can be accessed here:


